# Differences in lipid homeostasis and membrane lipid unsaturation confer differential tolerance to low temperatures in two *Cycas* species

**DOI:** 10.1186/s12870-021-03158-4

**Published:** 2021-08-16

**Authors:** Yanling Zheng, Yongqiong Yang, Meng Wang, Shijun Hu, Jianrong Wu, Zhixiang Yu

**Affiliations:** 1 Key Laboratory of State Forestry and Grassland Administration for Biodiversity Conservation in Southwest China, Southwest Forestry University, Kunming, 650233 Yunnan China; 2 Administration Bureau of Panzhihua Cycas National Nature Reserve, Panzhihua, 617000 Sichuan China

**Keywords:** Chlorophyll fluorescence parameters, Cold and freezing stress, *Cycas*, Endangered species, Lipidomics

## Abstract

**Background:**

*C. panzhihuaensis* is more tolerant to freezing than *C. bifida* but the mechanisms underlying the different freezing tolerance are unclear. Photosynthesis is one of the most temperature-sensitive processes. Lipids play important roles in membrane structure, signal transduction and energy storage, which are closely related to the stress responses of plants. In this study, the chlorophyll fluorescence parameters and lipid profiles of the two species were characterized to explore the changes in photosynthetic activity and lipid metabolism following low-temperature exposure and subsequent recovery.

**Results:**

Photosynthetic activity significantly decreased in *C. bifida* with the decrease of temperatures and reached zero after recovery. Photosynthetic activity, however, was little affected in *C. panzhihuaensis*. The lipid composition of *C. bifida* was more affected by cold and freezing treatments than *C. panzhihuaensis*. Compared with the control, the proportions of all the lipid categories recovered to the original level in *C. panzhihuaensis,* but the proportions of most lipid categories changed significantly in *C. bifida* after 3 d of recovery. In particular, the glycerophospholipids and prenol lipids degraded severely during the recovery period of *C. bifida*. Changes in acyl chain length and double bond index (DBI) occurred in more lipid classes immediately after low-temperature exposure in *C. panzhihuaensis* compare with those in *C. bifida*. DBI of the total main membrane lipids of *C. panzhihuaensis* was significantly higher than that of *C. bifida* following all temperature treatments.

**Conclusions:**

The results of chlorophyll fluorescence parameters confirmed that the freezing tolerance of *C. panzhihuaensis* was greater than that of *C. bifida*. The lipid metabolism of the two species had differential responses to low temperatures. The homeostasis and plastic adjustment of lipid metabolism and the higher level of DBI of the main membrane lipids may contribute to the greater tolerance of *C. panzhihuaensis* to low temperatures.

**Supplementary Information:**

The online version contains supplementary material available at 10.1186/s12870-021-03158-4.

## Background

Low temperature is a major threat to plants whose geographical distribution and development are limited. Many plant species in temperate regions can increase their freezing tolerance after exposure to low nonfreezing temperatures, a phenomenon known as cold acclimation [1]. However, some plant species distributed in tropical and subtropical areas may be sensitive to low temperatures above 0 °C [2]. It has been shown that membrane systems are particularly sensitive to low temperatures [3]. Freezing-induced extracellular ice formation could lead to membrane rupture due to mechanical stress and dehydration of the living cells [4]. Thylakoids possess the most abundant membranes in plant leaves where the light-dependent reactions of photosynthesis occur. Under low temperatures, the balance between light harvesting and light utilization for assimilation may be disrupted [5]. The excessive absorbed energy can lead to oxidative stress by overproducing reactive oxygen species (ROS) [6]. The chloroplast is proven to be the main site of ROS production and ROS attack under stress [7]. Therefore, photosynthesis is extremely sensitive to cold/freezing stress in plants [8]. However, some plant species have evolved adaptive mechanisms to minimize the negative effects of freezing temperatures [4]. Some molecular, metabolic and physiological characteristics are modulated to enhance plant freezing tolerance [4, 9].

Lipids function in membrane structure, signal transduction and energy storage. Lipid composition varies among plant species, tissues and membranes and is affected by the developmental stage and environmental conditions [10, 11]. Lipid metabolism is closely related to plant development and stress response of plants. Membranes are the primary sites of low-temperature injury in plants [12, 13]. Therefore, membrane properties such as integrity and temperature-compatible fluidity are important for maintaining plant functions and enhancing survival under low temperatures [14]. Glycerophospholipids and saccharolipids are the main membrane lipids. The unsaturation level and acyl chain length of these lipids affect the membrane fluidity [15]. To cope with the adverse effects of low temperature, lipid compositions are adjusted to increase membrane fluidity. The amounts of bilayer-stabilizing lipids such as phosphatidylcholine (PC) and digalactosyldiacylglycerol (DGDG) are also increased [15, 16]. In addition to membrane lipids, glycerolipids are involved in plant tolerance to low temperature due to their functions in intracellular homeostasis and energy balance [17, 18]. The conversion of diacylglycerol (DAG) to triacylglycerol (TAG) contributes to freezing tolerance of some plant species [3].

Cycads are considered to be living fossils due to their primitive features. Cycad study has evolutionary and adaptive implications in the context of global environmental changes. More than 60% of the known cycad species are in danger of extinction [19]. *Cycas* is the oldest genus of cycads and these species are restricted to the tropical and subtropical areas of Asia, Eastern Africa and Madagascar islands and the Australia Pacific islands [20, 21]. More than 20 species of *Cycas* occur in China, and all of them have been proposed as first-ranked plants for national protection [22]. *Cycas* species are considered to be sensitive to low temperature, but there are few studies on the adaptation of these species to cold and freezing temperatures.

*C. bifida* is one of the rarest cycads in China, and the distribution of this species is restricted to areas of Yunnan and Guangxi provinces. *C. panzhihuaensis* is endemic to the dry-hot valleys of the Jinsha River in southwest China. Its natural distribution is in the northernmost limit areas and at the highest altitude among the *Cycas* species. The natural distributions of *C. bifida* and *C. panzhihuaensis* in China are restricted to the subtropical zone. Previous research has showed that the freezing tolerance of *C. panzhihuaensis* is greater than that of *C. bifida* [23]. However, the responses of these species to low temperatures and subsequent recovery conditions are unclear. The goal of this study was to dissect the effects of cold, freezing and subsequent recovery on photosynthetic activities and lipid metabolism of *C. bifida* and *C. multipinnata*. The results can increase understanding of the freezing sensitivity and distribution of the two species.

## Results

### Changes in chlorophyll fluorescence parameters

Chlorophyll fluorescence is a non-invasive and highly sensitive probe used for monitoring the effects of environmental stresses on photosynthesis. Fv/Fm, Y(II) and rETR of *C. bifida* decreased significantly with the decrease of temperature, reaching the lowest level (near zero) after 3 d of recovery (Table 1). However, Y(NO) of *C. bifida* displayed an opposite trend, increasing by 323.21% for the recovered seedlings compared to the control. Y(NPQ) of *C. bifida* decreased significantly in seedlings subjected to freezing and recovery treatments, by 75.87 and 83.49%, respectively. qP and qN of *C. bifida* decreased significantly in seedlings subjected to freezing treatment, then qP increased to the control level and qN increased significantly following recovery. These data suggest that cold diminished the photosynthetic activity and freezing severely damaged the photosynthetic apparatus of *C. bifida*. Fv/Fm of *C. panzhihuaensis* decreased significantly in seedlings subjected to cold, freezing and recovery treatments, by 4.96, 5.54 and 5.07%, respectively. Y(NO) increased significantly but qN decreased significantly for seedlings subjected to freezing treatment. Except for Fv/Fm, Y(NO) and qN, all other parameters remained unchanged following low-temperature treatments. Both Y(II) and rETR of *C. panzhihuaensis* decreased significantly but Y(NPQ) increased significantly for recovered seedlings. Fv/Fm, Y(II) and rETR of *C. panzhihuaensis* were significantly higher than those of *C. bifida* after the various treatments. These results demonstrate that the photosynthesis of *C. panzhihuaensis* was only slightly affected by cold, and the photosynthetic apparatus was damaged by freezing to a lesser extent than that of *C. bifida*.

**Table 1:** Effects of low temperature treatments on chlorophyll fluorescence parameters of *C. bifida* and *C. panzhihuaensis*

Parameters	Species	Treatment			
		Control	Cold	Freezing	Recovery
Fv/Fm	*C. bifida*	0.826 ± 0.006a	0.782 ± 0.015b	0.717 ± 0.046c	0.001 ± 0.002d
	*C. panzhihuaensis*	0.857 ± 0.010a*	0.824 ± 0.004b*	0.819 ± 0.007b*	0.823 ± 0.009b*
Y(II)	*C. bifida*	0.462 ± 0.068a	0.325 ± 0.052b	0.152 ± 0.040c	0 ± 0d
	*C. panzhihuaensis*	0.465 ± 0.050a	0.421 ± 0.044ab*	0.427 ± 0.018ab*	0.383 ± 0.026b*
Y(NPQ)	*C. bifida*	0.315 ± 0.084a	0.321 ± 0.045a	0.076 ± 0.057b	0.052 ± 0.005b
	*C. panzhihuaensis*	0.260 ± 0.046b	0.288 ± 0.034ab	0.226 ± 0.030b*	0.313 ± 0.034a*
Y(NO)	*C. bifida*	0.224 ± 0.016d	0.354 ± 0.014c	0.776 ± 0.104b	0.948 ± 0.005a
	*C. panzhihuaensis*	0.275 ± 0.016b*	0.291 ± 0.039b*	0.348 ± 0.023a*	0.304 ± 0.013b*
qp	*C. bifida*	0.744 ± 0.044a	0.533 ± 0.061ab	0.241 ± 0.054b	0.8 ± 0.0447a
	*C. panzhihuaensis*	0.635 ± 0.048a*	0.644 ± 0.070a*	0.634 ± 0.019a*	0.588 ± 0.028a*
qN	*C. bifida*	0.682 ± 0.106b	0.596 ± 0.043b	0.134 ± 0.100c	0.987 ± 0.029a
	*C. panzhihuaensis*	0.561 ± 0.050a*	0.602 ± 0.054a	0.490 ± 0.051b*	0.612 ± 0.043a*
rETR	*C. bifida*	120.4 ± 17.501a	83.6 ± 13.390b	38 ± 9.925c	0 ± 0d
	*C. panzhihuaensis*	117.4 ± 12.740a	108.6 ± 11.127a*	108.6 ± 4.393a*	94.8 ± 6.686b*

Different letters in the same row indicate significant differences between treatments within species and * indicates significant difference between species within treatment (*P* < 0.05). Data are mean ± standard deviation (*n* = 4)

*Fv/Fm* The maximum quantum yield of photosystem II (PSII), *qP* Photochemical quenching coefficient, *qN* Non-photochemical quenching coefficient, *rETR* Relative electron transport rate, *Y(II)* Effective quantum yield of PS II, *Y(NO)* Non-regulated non-photochemical energy loss in PS II, *Y(NPQ)* Regulated non-photochemical energy loss in PS II

### Lipids profiling in the leaves of *C. bifida* and *C. panzhihuaensis*

**Fig. 1:**
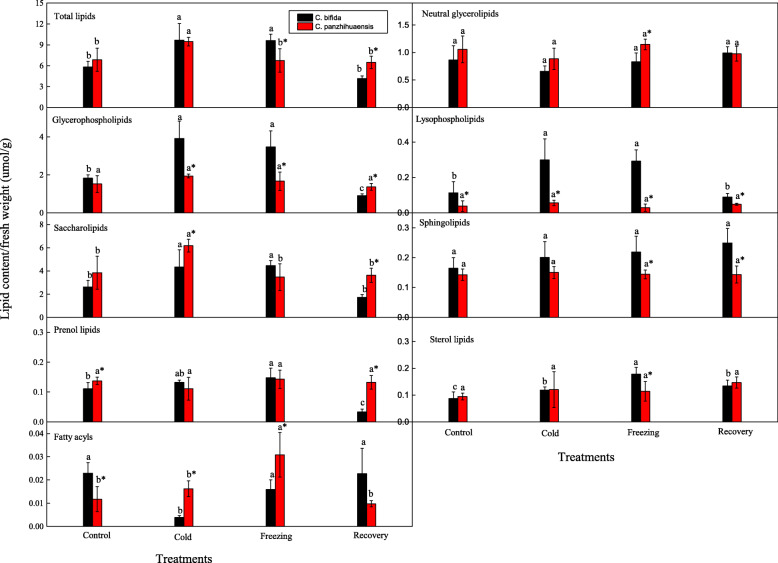
The content of each lipid category in *C. bifida* and *C. panzhihuaensis* treated at low temperatures. Different letters indicate significant differences between treatments within species and * indicates significant difference between species within treatment (*P* < 0.05). Data are mean ± standard deviation (*n* = 5)

A total of 26 lipid classes including 613 lipid species were identified from the leaves of *C. bifida* and *C. panzhihuaensis* (Table S1). The lipids contained two neutral glycerolipid classes, eight classes of glycerophospholipids (excluding lysophospholipids here), four of lysophospholipids, four of saccharolipids, four of sphingolipids, two of sterol lipids, one of prenol lipids (coenzyme Q) and one of fatty acyls (wax esters). The total lipid content of *C. bifida* was not significantly different from that of *C. panzhihuaensis*. Neutral glycerolipids, glycerophospholipids and saccharolipids were the main lipid categories for both the species, accounting for more than 90% of the total lipids (Table 2). The absolute content of neutral glycerolipids, glycerophospholipids and saccharolipids did not differ but the proportions of glycerophospholipids and saccharolipids differed significantly between the two species (Fig. 1; Table 2). The ratio of saccharolipids to phospholipids was significantly higher in *C. panzhihuaensis* (Table S2). Phosphatidic acid (PA), PC, phosphatidylethanolamine (PE), phosphatidylglycerol (PG) and phosphatidylinositol (PI) were the main glycerophospholipids. Monogalactosyldiacylglycerol (MGDG), DGDG and sulphoquinovosyldiacylglycerol (SQDG) were the main saccharolipids (Table 3).

**Table 2:** The proportion of each lipid category in *C. bifida* and *C. panzhihuaensis* treated at low temperatures

Lipid category	Species	Treatment			
		Control	Cold	Freezing	Recovery
		Lipid proportion (%)			
Neutral glycerolipids	*C. bifida*	15.50 ± 6.63b	7.11 ± 1.78c	8.76 ± 2.04c	23.86 ± 1.16a
	*C. panzhihuaensis*	16.72 ± 6.93a	9.35 ± 1.89b	17.81 ± 4.63a*	15.12 ± 0.80a*
Glycerophospholipids	*C. bifida*	31.09 ± 5.19b	40.77 ± 3.67a	35.90 ± 6.46ab	21.87 ± 2.38c
	*C. panzhihuaensis*	21.99 ± 1.03b*	20.58 ± 1.84b*	24.58 ± 1.64a*	21.11 ± 1.32b
Saccharolipids	*C. bifida*	44.69 ± 4.48a	44.02 ± 5.24a	46.47 ± 4.19a	41.47 ± 2.81a
	*C. panzhihuaensis*	54.86 ± 7.14b*	65.25 ± 1.91a*	50.55 ± 5.31b	55.87 ± 2.26b*
Lysophospholipids	*C. bifida*	1.92 ± 0.94a	3.06 ± 0.92a	3.06 ± 0.68a	2.15 ± 0.28a
	*C. panzhihuaensis*	0.51 ± 0.28a*	0.59 ± 0.14a*	0.43 ± 0.29a*	0.76 ± 0.15a*
Sphingolipids	*C. bifida*	2.89 ± 0.79b	2.25 ± 1.05b	2.27 ± 0.45b	6.03 ± 1.19a
	*C. panzhihuaensis*	2.17 ± 0.50a	1.60 ± 0.24a	2.25 ± 0.59a	2.27 ± 0.61a*
Sterol lipids	*C. bifida*	1.51 ± 0.39b	1.30 ± 0.39b	1.85 ± 0.22b	3.27 ± 0.73a
	*C. panzhihuaensis*	1.48 ± 0.52a	1.29 ± 0.75a	1.72 ± 0.47a	2.30 ± 0.43a*
Prenol lipids	*C. bifida*	1.97 ± 0.64a	1.44 ± 0.34a	1.53 ± 0.25a	0.81 ± 0.18b
	*C. panzhihuaensis*	2.08 ± 0.45a	1.17 ± 0.35b	2.17 ± 0.53a*	2.05 ± 0.18a*
Fatty acyls	*C. bifida*	0.40 ± 0.10a	0.04 ± 0.02b	0.17 ± 0.04b	0.55 ± 0.24a
	*C. panzhihuaensis*	0.19 ± 0.08b*	0.17 ± 0.03b*	0.51 ± 0.20a*	0.15 ± 0.04b*

Different letters in the same row indicate significant differences between treatments within species and * indicates significant difference between species within treatment (*P* < 0.05). Data are mean ± standard deviation (*n* = 5)

**Table 3:** The content of each main membrane lipid class in *C. bifida* and *C. panzhihuaensis* treated at low temperatures

Lipid class	Species	Treatment			
		Control	Cold	Freezing	Recovery
		Lipid content (μmol/g FW)			
		Control	Cold	Freezing	Recovery
PA	*C. bifida*	0.74 ± 0.41b	1.81 ± 0.59a	1.27 ± 0.30ab	0.26 ± 0.06b
	*C. panzhihuaensis*	0.30 ± 0.16a	0.42 ± 0.06a*	0.36 ± 0.05a*	0.35 ± 0.07a
PC	*C. bifida*	0.21 ± 0.09b	0.46 ± 0.17a	0.42 ± 0.08a	0.13 ± 0.02b
	*C. panzhihuaensis*	0.26 ± 0.15b	0.41 ± 0.01a	0.15 ± 0.02b*	0.22 ± 0.12b
PE	*C. bifida*	0.10 ± 0.01c	0.22 ± 0.04a	0.17 ± 0.02b	0.06 ± 0.00d
	*C. panzhihuaensis*	0.12 ± 0.06b	0.19 ± 0.04a	0.09 ± 0.02b*	0.10 ± 0.04b
PG	*C. bifida*	0.27 ± 0.09b	0.52 ± 0.17a	0.49 ± 0.05a	0.18 ± 0.02b
	*C. panzhihuaensis*	0.32 ± 0.04a	0.28 ± 0.04ab*	0.19 ± 0.03c*	0.25 ± 0.02b*
PI	*C. bifida*	0.45 ± 0.15b	0.85 ± 0.21ab	1.06 ± 0.04a	0.23 ± 0.05b
	*C. panzhihuaensis*	0.47 ± 0.10b	0.57 ± 0.08ab*	0.78 ± 0.30a	0.37 ± 0.08b*
PS	*C. bifida*	0.03 ± 0.01b	0.04 ± 0.01a	0.04 ± 0.00ab	0.02 ± 0.00c
	*C. panzhihuaensis*	0.03 ± 0.01a	0.04 ± 0.00a	0.04 ± 0.01a	0.04 ± 0.00a*
MGMG	*C. bifida*	0.04 ± 0.01b	0.07 ± 0.02a	0.07 ± 0.02a	0.04 ± 0.01b
	*C. panzhihuaensis*	0.08 ± 0.05b	0.17 ± 0.05a*	0.07 ± 0.02b	0.08 ± 0.02b*
MGDG	*C. bifida*	0.50 ± 0.10b	1.15 ± 0.57a	1.03 ± 0.22a	0.55 ± 0.10b
	*C. panzhihuaensis*	1.25 ± 0.76b	2.64 ± 0.35a*	0.65 ± 0.21b*	1.02 ± 0.46b
DGDG	*C. bifida*	0.56 ± 0.07c	1.13 ± 0.23b	1.36 ± 0.11a	0.09 ± 0.01d
	*C. panzhihuaensis*	0.80 ± 0.24b	1.39 ± 0.17a	0.84 ± 0.45b	0.84 ± 0.41b*
SQDG	*C. bifida*	1.53 ± 0.36b	1.99 ± 0.36a	2.00 ± 0.20a	1.04 ± 0.13c
	*C. panzhihuaensis*	1.72 ± 0.39a	1.98 ± 0.17a	1.91 ± 0.50a	1.69 ± 0.41a*
Total	*C. bifida*	4.46 ± 0.99b	8.25 ± 2.34a	7.94 ± 0.90a	2.63 ± 0.28b
	*C. panzhihuaensis*	5.37 ± 1.86b	8.12 ± 0.54a	5.14 ± 1.61b*	5.00 ± 0.76b*

Different letters in the same row indicate significant differences between treatments within species and * indicates significant difference between species within treatment (*P* < 0.05). Data are mean ± standard deviation (*n* = 5)

*DGDG* Digalactosyldiacylglycerol, *MGDG* Monogalactosyldiacylglycerol, *MGMG* monogalactosylmonoacylglycerol, *PA* Phosphatidic acid, *PC* Phosphatidylcholine, *PE* Phosphatidylethanolamine, *PG* Phosphatidylglycerol, *PI* Phosphatidylinositol, *PS* Phosphatidylserine, *SQDG* Sulphoquinovosyldiacylglycerol

### Changes in the composition of the lipid categories

The content of total lipids, glycerophospholipids, lysophospholipids, saccharolipids, prenol lipids and sterol lipids in *C. bifida* increased after the cold and freezing treatments. However, glycerophospholipids and prenol lipids decreased significantly and sterol lipids increased significantly after 3 d of recovery compared to those of the control (Fig. 1). The content of fatty acyls decreased significantly after cold treatment but recovered to the original level after freezing and recovery treatments. The ratio of saccharolipids to glycerophospholipids of *C. bifida* increased significantly after recovery treatments, which was mainly due to the severe degradation of the glycerophospholipids (Table S2). This suggests that the glycerophospholipids of *C. bifida* were more easily affected by freezing temperature than the saccharolipids. Compared to the control, the relative contents of all the lipid categories except saccharolipids, lysophospholipids and fatty acyls in *C. bifida* changed significantly after recovery (Table 2). This shows that the lipid metabolic balances of *C. bifida* were disturbed by low temperatures. For *C. panzhihuaensis*, the content of total lipids and saccharolipids increased significantly after cold treatment, and that of fatty acyls increased significantly after freezing treatment (Fig. 1). The ratio of saccharolipids to glycerophospholipids of *C. panzhihuaensis* increased significantly after cold treatment which was mainly due to the accumulation of saccharolipids (Table S2). Although the relative contents of neutral glycerolipids, glycerophospholipids, saccharolipids, prenol lipids and fatty acyls changed significantly following cold or freezing treatment, they all returned to the control level after recovery (Table 2). This suggests that the lipid metabolism of *C. panzhihuaensis* could positively respond to low-temperature stress and then recover to the normal state. It can also be concluded that freezing and subsequent thawing did not disturb the lipid metabolic system.

In a comparison of the two species, the contents of glycerophospholipids and lysophospholipids were significantly lower and those of saccharolipids and fatty acyls were significantly higher in *C. panzhihuaensis* after cold treatment. The contents of total lipids, glycerophospholipids, lysophospholipids, sphingolipids and sterol lipids were significantly lower and those of neutral glycerolipids and fatty acyls were significantly higher in *C. panzhihuaensis* following freezing treatment. After recovery treatment, the contents of total lipids, glycerophospholipids, saccharolipids and prenol lipids were significantly higher and those of lysophospholipids and sphingolipids were significantly lower in *C. panzhihuaensis*.

### Changes in the composition of main lipid classes and lipid species of glycerophospholipids and saccharolipids

**Fig. 2:**
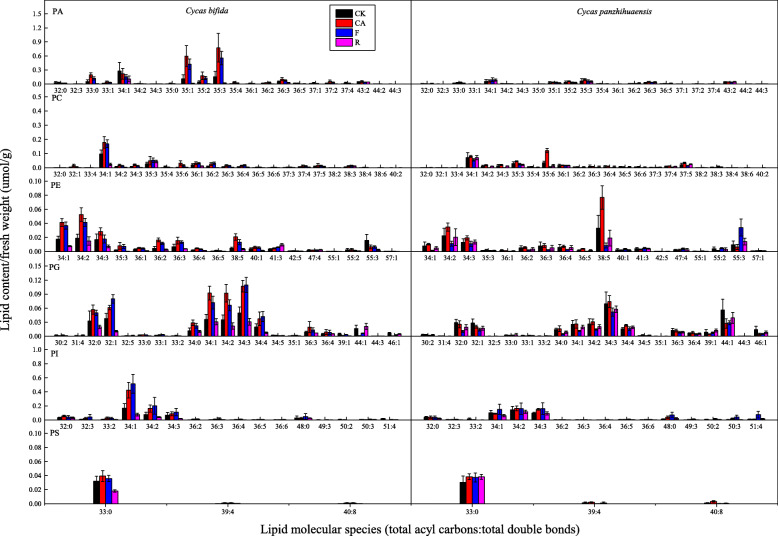
Changes in lipid molecular species of glycerophospholipids in *C. bifida* and *C. panzhihuaensis* treated at low temperatures. Data are mean ± standard deviation (*n* = 5). PA: Phosphatidic acid; PC: Phosphatidylcholine; PE: Phosphatidylethanolamine; PG: Phosphatidylglycerol; PI: Phosphatidylinositol; PS: Phosphatidylserine

Phosphatidylinositol phosphate (PIP) and cardiolipin (CL) were also detected, but their levels were extremely low (Table S3). Therefore, these two lipid classes were not analyzed separately but included in the analysis of total glycerophospholipids. PA, PC, PE, PG, PI and PS contents of *C. bifida* increased significantly after cold and/or freezing treatments (Table 3). However, PA, PC, PG and PI contents recovered to the original level and PE and PS contents decreased significantly after 3 d of recovery. Compared to the control, PC and PE levels in *C. panzhihuaensis* increased significantly after cold treatment (Table 3). PG content decreased significantly but PI content increased significantly after freezing treatment. After recovery, PG content increased significantly compared to that of the freezing-treated seedlings but did not reach the original level. PA, PG and PI levels in *C. panzhihuaensis* were significantly lower than levels in *C. bifida* after cold treatment. PA, PC, PE and PG contents of *C. panzhihuaensis* were significantly lower than those of *C. bifida* after freezing treatment. After recovery, PG, PI and PS contents of *C. panzhihuaensis* were significantly higher than those of *C. bifida*. Among the main lipid species of the several lipid classes, there were significantly different responses of the two species to various treatments in all the PA species except 32:0, 34:1, 34:2, 34:3 and 43:2; all the PC species except 35:3, 35:4, 37:3, 37:4 and 37:5; all the PE species except 36:4 and 47:4; all the PG species except 30:2, 34:5, 36:3, 36:4, 44:1 and 46:1; PI species 33:2, 34:1, 36:3, 49:3, 50:2, 50:3 and 51:4; PS species 33:0, 39:4 and 40:8 (Fig. 2).

**Fig. 3:**
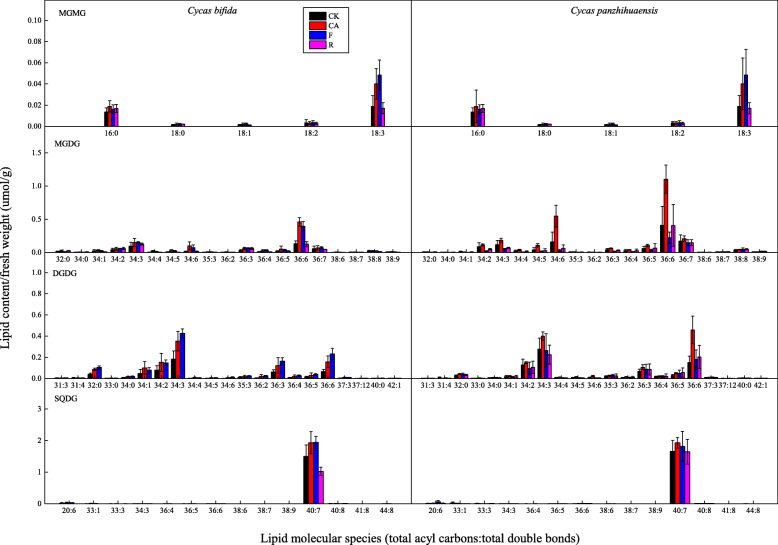
Changes in lipid molecular species of saccharolipids in *C. bifida* and *C. panzhihuaensis* treated at low temperatures. Data are mean ± standard deviation (*n* = 5). DGDG: Digalactosyldiacylglycerol; MGDG: Monogalactosyldiacylglycerol; MGMG: monogalactosylmonoacylglycerol; SQDG: Sulphoquinovosyldiacylglycerol

Compared to those of the control, the content of each class of saccharolipids in *C. bifida* increased significantly after cold and freezing treatments (Table 3). Monogalactosylmonoacylglycerol (MGMG) and MGDG then returned to the original level but DGDG and SQDG decreased, by 83.93 and 32.03% respectively, after 3 d of recovery compared to the control. These results show that the equilibrium of saccharolipid metabolism of *C. bifida* was disrupted due to the degradation of DGDG and SQDG at the recovery stage. For *C. panzhihuaensis*, SQDG content did not vary among the control and different treatments (Table 3). MGMG, MGDG and DGDG only responded to cold treatment, increasing by 112.5, 111.2 and 73.75%, respectively. Compared to those of *C. bifida*, both MGMG and MGDG contents of *C. panzhihuaensis* were significantly higher in the cold-treated seedlings; MGDG content was significantly lower for the freezing-treated seedlings; MGMG, DGDG and SQDG content were significantly higher for the recovered seedlings. Among the main lipid species of the several lipid classes, there were significantly different responses of the two species to various treatments in MGMG species 18:3; MGDG species 34:2, 34:3, 34:4, 34:5, 34:6, 35:3, 36:2, 36:3, 36:4, 36:6 and 38:6; all the DGDG species except 31:3, 35:3, 36:5, 40:0 and 42:1; SQDG species 36:6, 38:7, 38:9, 41:8 and 44:8 (Fig. 3).

### Changes in the ACL and DBI of glycerophospholipids and saccharolipids

The ACL of total glycerophospholipids, PE and PI of *C. bifida* was not affected by the treatments (Table 4). The ACL of PA and PC was not affected by low temperature but increased significantly after recovery. The ACL of PG decreased significantly after low-temperature treatments but then increased to the control level after recovery. The ACL of PS increased significantly after low-temperature and recovery treatments. *C. panzhihuaensis* had significantly different responses to various treatments compared to *C. bifida* in the ACL of PC, PE, PI and PS (Table 4). The ACL of PA and PE decreased and that of PS increased significantly after cold treatments. The ACL increased significantly for PC, PE, PI and total glycerophospholipids and decreased for PA and PS after freezing treatments. Compared to those in *C. bifida*, the ACL of most glycerophospholipid classes in *C. panzhihauensis* was higher following cold or/and freezing treatments but that of PS was lower following freezing. The ACL of total glycerophospholipids of *C. panzhihuaensis* was significantly higher than that of *C. bifida* after low-temperature treatments (Table 4).

**Table 4:** Acyl chain length (ACL) of each main membrane lipid class in *C. bifida* and *C. panzhihuaensis* treated at low temperatures

Lipid class	Species		Treatment		
		Control	Cold	Freezing	Recovery
		ACL			
PA	*C. bifida*	35.26 ± 0.67b	35.03 ± 0.10b	35.08 ± 0.08b	35.91 ± 0.51a
	*C. panzhihuaensis*	36.36 ± 0.62a*	35.73 ± 0.16b*	35.70 ± 0.21b*	36.00 ± 0.32ab
PC	*C. bifida*	34.99 ± 0.27b	34.89 ± 0.11b	34.99 ± 0.06b	35.50 ± 0.09a
	*C. panzhihuaensis*	35.13 ± 0.07b	35.11 ± 0.04b*	35.33 ± 0.10a*	35.12 ± 0.17b*
PE	*C. bifida*	39.20 ± 4.61a	36.26 ± 0.37a	36.37 ± 0.16a	37.61 ± 0.98a
	*C. panzhihuaensis*	39.33 ± 2.02b	37.07 ± 0.81c	44.68 ± 1.23a*	40.04 ± 1.75b*
PG	*C. bifida*	34.73 ± 0.78a	33.64 ± 0.19b	33.77 ± 0.07b	35.28 ± 0.55a
	*C. panzhihuaensis*	36.20 ± 1.43a	35.06 ± 0.58a*	35.93 ± 0.22a*	36.02 ± 0.65a
PI	*C. bifida*	35.85 ± 1.97ab	34.41 ± 0.40b	34.96 ± 0.63ab	36.51 ± 0.25a
	*C. panzhihuaensis*	35.21 ± 0.83b	36.02 ± 0.54b*	38.27 ± 0.47a*	36.06 ± 0.72b
PS	*C. bifida*	33.11 ± 0.02b	33.40 ± 0.11a	33.40 ± 0.18a	33.32 ± 0.04a
	*C. panzhihuaensis*	33.44 ± 0.28b	33.82 ± 0.15a*	33.10 ± 0.03c*	33.29 ± 0.25bc
**Phospholipids**	***C. bifida***	**36.04 ± 1.83a**	**34.81 ± 0.12a**	**35.03 ± 0.22a**	**36.43 ± 0.48a**
	***C. panzhihuaensis***	**36.25 ± 0.10b**	36.03 ± 0.24b*	38.06 ± 0.52a*	**36.49 ± 0.47b**
MGMG	*C. bifida*	17.23 ± 0.34a	17.41 ± 0.42a	17.50 ± 0.22a	17.15 ± 0.08a
	*C. panzhihuaensis*	17.41 ± 0.12b	17.70 ± 0.14a	17.49 ± 0.12ab	17.64 ± 0.24a
MGDG	*C. bifida*	35.22 ± 0.53a	35.19 ± 0.16a	35.30 ± 0.07a	35.04 ± 0.07a
	*C. panzhihuaensis*	35.41 ± 0.25b	35.26 ± 0.05b	35.78 ± 0.03a*	35.66 ± 0.07a*
DGDG	*C. bifida*	34.54 ± 0.23a	34.43 ± 0.24a	34.63 ± 0.18a	33.57 ± 0.46b
	*C. panzhihuaensis*	34.71 ± 0.07c	34.96 ± 0.05a*	34.87 ± 0.02b*	34.92 ± 0.10ab*
SQDG	*C. bifida*	39.68 ± 0.36a	39.69 ± 0.30a	39.61 ± 0.04a	39.93 ± 0.01a
	*C. panzhihuaensis*	39.71 ± 0.11a	39.77 ± 0.11a	39.29 ± 0.30b	39.76 ± 0.06a*
**Saccharolipids**	***C. bifida***	**37.39 ± 0.29ab**	**36.94 ± 0.57b**	**36.75 ± 0.23b**	**37.52 ± 0.17a**
	***C. panzhihuaensis***	**36.91 ± 0.42a**	36.19 ± 0.27b*	**37.20 ± 0.38a**	**37.05 ± 0.71a**
**Total**	***C. bifida***	**36.79 ± 0.75a**	**35.89 ± 0.20b**	**35.99 ± 0.11b**	**37.13 ± 0.23a**
	***C. panzhihuaensis***	**36.73 ± 0.57b**	**36.15 ± 0.22b**	37.47 ± 0.28a*	**36.89 ± 0.64ab**

ACL = (∑[n × mol % lipid])/100, where n is the number of acyl carbons in each lipid molecule. Different letters in the same row indicate significant differences between treatments within species and * indicates significant difference between species within treatment (*P* < 0.05). Data are mean ± standard deviation (*n* = 5)

*DGDG* Digalactosyldiacylglycerol, *MGDG* Monogalactosyldiacylglycerol, *MGMG* monogalactosylmonoacylglycerol, *PA* Phosphatidic acid, *PC* Phosphatidylcholine, *PE* Phosphatidylethanolamine, *PG* Phosphatidylglycerol, *PI* Phosphatidylinositol, *PS* Phosphatidylserine, *SQDG* Sulphoquinovosyldiacylglycerol

Except for decreasing significantly in DGDG after recovery, the ACL of other classes of saccharolipids in *C. bifida* did not vary among the treatments (Table 4). The ACL of total saccharolipids of *C. bifida* showed no significant change after various treatments. For *C. panzhihauensis*, the ACL of MGMG, MGDG and DGDG increased significantly after cold or/and freezing treatment and that of SQDG decreased significantly after freezing treatment (Table 4). Except for SQDG, the ACL of all the classes of saccharolipids increased significantly after 3 d of recovery, compared to that of the control. The ACL of total saccharolipids in *C. panzhihauensis* showed a significant decrease after cold treatment, which was significantly lower than that of *C. bifida*. The ACL of the total main membrane lipids (glycerophospholipids and saccharolipids) decreased significantly after low-temperature treatments in *C.bifida* and increased significantly after freezing treatment in *C. panzhihuaensis*, being significantly higher than that of *C. bifida*.

Except for increasing significantly in PS after low-temperature treatments, DBI of every glycerophospholipid class and total glycerophospholipids in *C. bifida* did not change (Table 5). For *C. panzhihauensis*, DBI of all the glycerophospholipid classes increased significantly after cold treatment, and those of PG and PI also increased significantly after freezing and recovery treatments, but the unsaturation level of PS decreased significantly after freezing treatment. The DBI of total glycerophospholipids in *C. panzhihauensis* increased significantly after cold treatment. Compared to those of *C. bifida*, the DBI of all the glycerophospholipid classes except PG of *C. panzhihuaensis* was significantly higher in cold-treated seedlings, which of PA, PE, PG and PI were significantly higher in freezing-treated and recovered seedlings (Table 5). The DBI and DBI/ACL of total glycerophospholipids of *C. panzhihuaensis* were significantly higher than those of *C. bifida* after various treatments (Table 5; Table S4).

**Table 5:** Double bond index (DBI) of each main membrane lipid class in *C. bifida* and *C. panzhihuaensis* treated at low temperatures

Lipid class	Species	Treatment			
		Control	Cold	Freezing	Recovery
		DBI			
PA	*C. bifida*	1.57 ± 0.40a	1.48 ± 0.09a	1.53 ± 0.05a	1.60 ± 0.23a
	*C. panzhihuaensis*	1.87 ± 0.12b	2.02 ± 0.13a*	1.81 ± 0.07b*	1.95 ± 0.11ab*
PC	*C. bifida*	2.03 ± 0.30a	2.26 ± 0.32a	2.19 ± 0.21a	2.70 ± 0.35a
	*C. panzhihuaensis*	2.84 ± 0.37b*	3.69 ± 0.14a*	2.43 ± 0.16b	2.55 ± 0.49b
PE	*C. bifida*	2.43 ± 0.38a	2.40 ± 0.22a	2.34 ± 0.08a	2.57 ± 0.09a
	*C. panzhihuaensis*	3.08 ± 0.23b*	3.53 ± 0.22a*	2.92 ± 0.10b*	2.98 ± 0.29b*
PG	*C. bifida*	1.74 ± 0.23a	1.75 ± 0.19a	1.82 ± 0.10a	1.71 ± 0.06a
	*C. panzhihuaensis*	1.69 ± 0.18c	1.97 ± 0.09ab	2.02 ± 0.03a*	1.85 ± 0.06b*
PI	*C. bifida*	1.65 ± 0.33a	1.51 ± 0.08a	1.59 ± 0.06a	1.48 ± 0.06a
	*C. panzhihuaensis*	1.94 ± 0.08b	2.05 ± 0.08a*	2.09 ± 0.06a*	2.06 ± 0.04a*
PS	*C. bifida*	0.10 ± 0.02b	0.37 ± 0.13a	0.36 ± 0.16a	0.29 ± 0.04ab
	*C. panzhihuaensis*	0.39 ± 0.25b	0.80 ± 0.18a*	0.10 ± 0.04c*	0.25 ± 0.20bc
**Phospholipids**	***C. bifida***	**1.70 ± 0.35a**	**1.67 ± 0.12a**	**1.72 ± 0.10a**	**1.80 ± 0.06a**
	***C. panzhihuaensis***	**2.10 ± 0.19b**	2.50 ± 0.14a*	2.06 ± 0.05b*	2.13 ± 0.20b*
MGMG	*C. bifida*	1.58 ± 0.49a	1.92 ± 0.67a	2.03 ± 0.39a	1.44 ± 0.13a
	*C. panzhihuaensis*	1.93 ± 0.18b	2.44 ± 0.22a	2.02 ± 0.23b	2.29 ± 0.40ab*
MGDG	*C. bifida*	4.53 ± 1.07a	4.70 ± 0.41a	4.80 ± 0.26a	4.08 ± 0.14a
	*C. panzhihuaensis*	5.27 ± 0.13c	5.46 ± 0.08b*	5.77 ± 0.08a*	5.51 ± 0.13b*
DGDG	*C. bifida*	2.93 ± 0.44b	2.82 ± 0.26b	3.11 ± 0.33b	3.69 ± 0.12a
	*C. panzhihuaensis*	3.35 ± 0.18c	3.88 ± 0.18a*	3.49 ± 0.06bc*	3.56 ± 0.13b
SQDG	*C. bifida*	6.96 ± 0.03a	6.95 ± 0.03a	6.95 ± 0.01a	6.97 ± 0.00a
	*C. panzhihuaensis*	6.89 ± 0.09a	6.95 ± 0.03a	6.94 ± 0.01a	6.95 ± 0.01a*
**Saccharolipids**	***C. bifida***	**5.56 ± 0.34ab**	**5.32 ± 0.24b**	**5.22 ± 0.06b**	**5.75 ± 0.09a**
	***C. panzhihuaensis***	**5.55 ± 0.12a**	**5.51 ± 0.02a**	5.84 ± 0.17a*	**5.70 ± 0.36a**
**Total**	***C. bifida***	**3.99 ± 0.46ab**	**3.55 ± 0.16b**	**3.69 ± 0.30b**	**4.39 ± 0.17a**
	***C. panzhihuaensis***	**4.55 ± 0.07b**	4.79 ± 0.09a*	4.59 ± 0.06b*	4.72 ± 0.20ab*

DBI = Σ(N× mol% lipid)/100, where N is the number of double bonds in each lipid molecule. Different letters in the same row indicate significant differences between treatments within species and * indicates significant difference between species within treatment (*P* < 0.05). Data are mean ± standard deviation (*n* = 5)

*DGDG* Digalactosyldiacylglycerol, *MGDG* Monogalactosyldiacylglycerol, *MGMG* monogalactosylmonoacylglycerol, *PA* Phosphatidic acid, *PC* Phosphatidylcholine, *PE* Phosphatidylethanolamine, *PG* Phosphatidylglycerol, *PI* Phosphatidylinositol, *PS* Phosphatidylserine, *SQDG* Sulphoquinovosyldiacylglycerol

Except that DBI of DGDG increased significantly after recovery, the DBI of every class of saccharolipids and the total saccharolipids in *C. bifida* were not affected by low temperatures. For *C. panzhihuaensis*, the DBI of MGDG in low-temperature-treated seedlings and those of MGMG and DGDG in cold-treated seedlings increased significantly compared to the control. Compared to those of *C. bifida*, the DBI of MGDG and DGDG in low-temperature-treated seedlings of *C. panzhihuaensis* and that of total saccharolipids in freezing-treated seedlings were significantly higher. The DBI and DBI/ACL of total main membrane lipids were significantly higher in *C. panzhihuaensis* than those in *C. bifida* following low temperature and recovery treatments (Table 5; Table S4).

## Discussion

Low temperature is a key factor limiting the introduction of tropical and subtropical plants, including cycads, to higher latitudes and altitudes. We previously showed that the freezing tolerance of *C. panzhihuaensis* was greater than that of *C. bifida* [23]. However, a systematic and in-depth study of the adaptation of the two species to low temperatures is still lacking. Lipids play key roles in diverse cellular processes and lipid metabolism is closely related to freezing tolerance of some plants [3, 24]. However, how lipids adjust under low temperature to regulate the tolerance of *C. panzhihuaensis* and *C. bifida* to low-temperature stress is not understood. Photosynthesis is a highly temperature-sensitive process [8]. Therefore, it is often used to reflect the adaptability of plants to temperature change. In the present study, the chlorophyll fluorescence parameters and lipid profiles of the two species subjected to cold, freezing and subsequent recovery were characterized.

### The reduction and loss of photosynthetic activities

Chlorophyll fluorescence can sensitively reflect the physiological status of plants. The significant decrease of Fv/Fm, Y(II) and rETR and significant increase of Y(NO) with the decrease of temperatures in *C. bifida* demonstrate that photosynthetic activities of this species were affected, particularly severely by the freezing treatment. Fv/Fm, Y(II) and rETR reached to zero after 3 d of recovery and indicated that the photosynthetic apparatus of *C. bifida* was severely damaged and unable to recover. For *C. panzhihuaensis*, only Fv/Fm of the cold-treated seedlings decreased by 3.85% and Fv/Fm, Y(II) and rETR of the recovered seedlings decreased only by 3.97, 17.63 and 19.25%, respectively, compared to those of the control. These values show that *C. panzhihuaensis* was relatively little affected by cold and freezing temperatures in comparison with *C. bifida*. The damaging effects of freezing on plant morphology might not appear immediately after treatments. They can, however, be more obvious after a period of recovery. We observed that freezing-treated leaves of *C. bifida* gradually became yellow and dry but those of *C. panzhihuaensis* remained green after 10 d of recovery. These results confirm that *C. bifida* is more sensitive to low temperatures than *C. panzhihuaensis*.

### The changes in composition of the lipid categories

The metabolism of neutral glycerolipids is affected by low temperature and is related to plant tolerance to low temperatures [24]. For example, DAG and TAG accumulated and the DAG/TAG ratio decreased under freezing in *Arabidopsis* [16, 24]. The accumulation of TAG due to the conversion of DAG can contribute to the freezing tolerance of plants [3, 24]. However, in this study the neutral glycerolipid content remained unchanged, and the DAG-TAG ratios of the treated seedlings were not significantly different from the control for either species (Fig. 1; Table S5). These results show that the freezing sensitivity of the two species had little relation to the neutral glycerolipid metabolism under freezing conditions. Glycerophospholipids and saccharolipids are the main extraplastidic and plastidic membrane lipids, respectively, in plants. Some data indicate that the two categories of lipids can degrade under low temperatures [15, 25]. However, the glycerophospholipids and saccharolipids contents increased significantly after cold and freezing treatments in *C. bifida* and saccharolipids increased after cold treatment in *C. panzhihuaensis*. The sources of these accumulated lipids under low temperatures are not clear. The metabolic pathways of carbohydrates and lipids undergo cross talk to regulate energy homeostasis [26], but whether the increased lipids are due to the conversion of the stored carbohydrates requires clarification. For all of the treatments, the saccharolipids/glycerophospholipids ratio of *C. panzhihuaensis* was always significantly higher than that of *C. bifida* (Table S2). It is unclear if this is related to the higher freezing tolerance of *C. panzhihuaensis*.

Except that the saccharolipid and fatty acyl content increased significantly following cold and freezing treatments, respectively, the absolute contents of all the lipid categories of *C. panzhihuaensis* did not change after the various treatments (Fig. 1). For *C. bifida*, the absolute contents of all the lipid categories except neutral glycerolipids and sphingolipids varied with the treatments to different extent (Fig. 1). These results demonstrate that the lipid metabolism of *C. bifida* was more affected by cold and freezing treatments than *C. panzhihuaensis*. The proportion of some lipid categories changed after low-temperature treatments in both *C. bifida* and *C. panzhihuaensis* (Table 2). However, the proportions of all the lipid categories recovered to the original level after 3 d of recovery for *C. panzhihuaensis*, which of most lipid categories changed significantly for *C. bifida* in comparison with the control. This showed the plastic adjustment of lipid metabolism in *C. panzhihauensis*, which might be related to the greater tolerance of the species to low temperature. Phospholipids are major structural components of cell membranes and are involved in signal transduction and energy storage [27]. Prenol lipids (coenzyme Q here) are essential for energy metabolism in the electron transport system and also function as antioxidants within membrane systems [28]. The disorders of lipid metabolism after recovery such as the degradation of phospholipids and prenol lipids might contribute to the ultimate death of aboveground parts of *C. bifida* seedlings.

Lysophospholipids, sphingolipids and sterols are structural components of membranes and also important signaling molecules involved in plant development and environmental responses [29–31]. The accumulation of lysophospholipids and sphingolipids under stress might be detrimental to the cells [32, 33]. Besides sterol contents, the contents of lysophospholipids and sphingolipids of *C. bifida* were significantly higher than those of *C. panzhihuaensis*. Whether the differential tolerance of the two species to freezing is related to their different contents and change patterns of these lipids needs further study. Cuticular waxes are the primary structures of the cuticle and play crucial roles in plant defense against biotic and abiotic stress including drought and frost [34]. The significantly higher content of wax esters of *C. panzhihuaensis* following cold and freezing treatments might contribute to its higher freezing tolerance than *C. bifida*.

### Changes in composition of the main lipid classes and lipid species of glycerophospholipids and saccharolipids

PA can form nonbilayer lipid structures with MGDG or DAG during low temperature, disrupting the integrity of cell membrane [35, 36]. Some studies showed that PA content increased dramatically under stresses such as freezing [15, 35]. PA content remained unchanged in *C. panzhihuaensis* after various treatments but increased by 144.59 and 71.62% in *C. bifida* after cold and freezing treatments, respectively (Table 3). The maintenance of PA content in *C. panzhihuaensis* was conducive to keep the membrane stability, but the increase of PA in *C. bifida* after low-temperature treatments might pose a potential threat to membrane integrity. The significantly lower level of PA after cold and freezing treatments might confer higher freezing tolerance to *C. panzhihuaensis*.

The glycerophospholipid composition of various plant species presented different responses to stresses [11, 15, 37]. For *C. bifida*, the content of each glycerophospholipid class increased significantly after cold or/and freezing treatment but the PE, PS and total glycerophospholipid contents decreased significantly after 3 d of recovery (Fig. 1; Table 3). These results suggest that glycerophospholipid metabolism of *C. bifida* was dramatically affected by low temperature and the membrane was severely damaged following freezing treatment. For *C. panzhihuaensis*, different classes of glycerophospholipids showed different change trends following low-temperatute treatments. However, all the classes except for PG recovered to the original level. This demonstrated that PG was more sensitive to freezing temperature than other phospholipids in *C. panzhihuaensis*. Other studies have suggested that the level of high-melting-point PG molecules such as 32:0 and 32:1 is related to the sensitivity of plants to low temperatures [38, 39]. The high-melting-point PG molecules were much lower in *C. panzhihuaensis* and showed a decrease and an increase after low temperature for *C. panzhihuaensis* and *C. bifida*, respectively (Fig. 2). This might be a factor involved in the freezing tolerance difference between the two species.

Saccharolipids are main lipids of the chloroplast envelope and thylakoid membrane, which play key roles in the photosynthetic process [40]. These lipids are likely to be degraded under some stresses [11, 41]. However, all the classes of saccharolipids in *C. bifida* increased significantly after low temperature treatments and all with the exception of DGDG and SQDG recovered to the control level after recovery (Table 3). The increase of saccharolipids after low temperatures suggests that seedlings of *C. bifida* positively resist the adverse effects of cold and freezing stresses on photosynthetic apparatus by stabilizing plastidic membranes. High DGDG/MGDG can be more conducive to maintaining bilayer membrane structure [16, 40]. The substantial degradation of DGDG during post-freezing recovery indicates that the membrane integrity of *C. bifida* was damaged following freezing temperature. This is consistent with the results of chlorophyll fluorescence parameters, which showed the severe loss of photosynthetic activity of *C. bifida*. In contrast, the saccharolipids of *C. panzhihuaensis* including MGMG, MGDG and DGDG only responded to cold treatment. This might be an adaptive mechanism of *C. panzhihuaensis* to cold temperature, but the relevance of these findings with the freezing tolerance of the species requires further study. MGDG and DGDG are the main components of saccharolipids in most plants [10, 42]. However, the SQDG contents of *C. bifida* and *C. panzhihuaensis* accounted for 58.21 and 44.69% of the total saccharolipids, respectively, surpassing the MGDG and DGDG contents (Fig. 1; Table 3). A Similar phenomenon was found in some algae and lichens [43, 44]. SQDG was also found in cyanobacteria and non-photosynthetic bacteria [43]. The high content of SQDG in the two *Cycas* species might be related to the highly diverse endophytic microbiome such as cyanobacteria and actinomycetes [45, 46]. It might also result from the adaptation of these species to nutrient-poor environments as P-starvation could promote SQDG accumulation [47].

### The changes in ACL and DBI of glycerophospholipids and saccharolipids

ACL and DBI are important determinants of membrane fluidity, which are related to the development and environmental adaptability of plants [15]. The decrease of ACL and increase of DBI under low temperature enable the membranes to be more fluid, which contributes to plant tolerance to low temperatures [15]. The ACL and DBI of different classes of glycerophospholipids and saccharolipids showed different change trends following various treatments for each species. Among the glycerophospholipids, PG is the major component in chloroplast membranes. ACL of PG in *C. bifida* decreased significantly and DBI of PG in *C. panzhihuaensis* increased significantly after low-temperature treatments. This suggests that the two species adopt different strategies to increase the membrane fluidity. Our previous study suggested that ACL of PS was related to the plant lifespan, which can be accelerated by senescence but ceased to increase in plants near death [48]. In the present study, ACL of PS increased significantly after low temperatures and did not return to the control level after 3 d of recovery in *C. bifida*. ACL of PS varied with the decrease of temperature but recovered to the control level subsequently in *C. panzhihuaensis*. This implies that freezing-treated seedlings of *C. bifida* might gradually lose viability, while those of *C. panzhihuaensis* were not severely damaged.

In general, the ACL and DBI changes occurred in more lipid classes immediately after low temperatures in *C. panzhihuaensis* than those in *C. bifida* (Tables 4 and 5). The ACL and DBI of glycerophospholipids and saccharolipids remained unchanged in *C. bifida*. However, ACL and DBI of glycerophospholipids and ACL of saccharolipids responded to cold or freezing treatment, and they all recovered to the original level subsequently in *C. panzhihuaensis* (Tables 4 and 5). These results show that *C. panzhihuaensis* can better adjust the membrane fluidity to respond to the decreasing temperature. Based on the higher level of DBI/ACL (Table S4), the higher level of DBI of total glycerophospholipids could maintain a higher fluidity of extraplastidic membranes under low temperature in *C. panzhihuaensis*, although the ACL was significantly higher*.* For total saccharolipids, the ACL of *C. panzhihuaensis* was shorter after cold temperature and DBI was higher after freezing temperature compared to those of *C. bifida*. This could enable the seedlings of *C. panzhihauensis* to obtain more fluidity of plastidic membranes which are apt to be damaged under low temperature. The results were consistent with the chlorophyll fluorescence parameters that photosynthetic activity following treatments severely was lost in *C. bifida* but little changed in *C. panzhihuaensis*. The DBI of the total main membrane lipids of *C. panzhihuaensis* was significantly higher than that of *C. bifida* following all the treatments. The higher level of DBI after low temperature treatments might contribute to the higher freezing tolerance of *C. panzhihuaensis*.

## Conclusions

The photosynthetic activity of *C. bifida* was more severely affected by low temperature than that of *C. panzhihuaensis*. The differential effects of freezing temperature were more obvious after 3 d of recovery. Seedlings of *C. bifida* lost almost all photosynthetic capacity, but *C. panzhihuaensis* seedlings were little affected. The results confirm previous work demonstrating that the freezing tolerance of *C. panzhihuaensis* was higher than that of *C. bifida*. The lipid composition of *C. bifida* was also more affected by cold and freezing treatments than *C. panzhihuaensis*. The proportions of all the lipid categories recovered to the original level for *C. panzhihuaensis*, but those of most lipid categories were changed significantly in *C. bifida* after 3 d of recovery. The homeostasis and plastic adjustment of lipid metabolism in *C. panzhihauensis* might be related to the greater tolerance of the species to low temperature than *C. bifida*. However, the severe degradation of glycerophospholipids and prenol lipids might be an important determinant of seedling death during the recovery period for *C. bifida*. The changes of ACL and DBI occurred in more lipid classes immediately after low temperatures in *C. panzhihuaensis* than those in *C. bifida*. The higher level of DBI of the main membrane lipids following low-temperature treatments might contribute to the higher freezing tolerance of *C. panzhihuaensis*. Lipid metabolism underwent different changes in seedlings of *C. panzhihuaensis* and *C. bifida*, which might be involved in the differential tolerance of the two species to low temperatures.

## Methods

### Plant materials and treatments

Seeds of *C. panzhihuaensis* were collected in Panzhihua. This was approved by the Administration Bureau of Panzhihua Cycas National Nature Reserve, Sichuan province. Seeds of *C. bifida* were collected from horticultural sources in Gejiu, Yunnan province. Seed collection was permitted by private land owners. The germinated four-year-old seedlings of *C. bifida* and *C. panzhihuaensis* were grown in a greenhouse in Southwest Forestry University and used to conduct the experiments. The seedlings of the two species were identified based on morphological characteristics by Shuangzhi Li, a taxonomist expert at Southwest Forestry University. Voucher specimens of *C. panzhihuaensis* (No. ZYL-001) and *C. bifida* (No. ZYL-002) were prepared and deposited in the Southwest Forestry University. The average temperature of the greenhouse was about 30 ± 1 °C, and the daytime photosynthetic photon flux density was about 250–300 μmol m^− 2^ s^− 1^. All of the seedlings were planted in plastic pots containing humus and laterite soil (1:1 v/v). For cold treatment, seedlings were transferred to artificial chambers at a constant temperature of 4 °C for 3 d. The freezing and thaw treatments were conducted as described by Zhang et al. [49] and Arisz et al. [3] with some modifications. For the freezing treatment, the cold-treated seedlings were put in a programmable temperature incubator set at 0 °C. Freezing was initiated by spraying ice cold water, and the temperature of the chamber was lowered at a rate of 2 °C h^− 1^ until − 5 °C was reached. They were then maintained for 1.5 h at − 5 °C, after which the temperature was increased up to 4 °C at a rate of 2 °C h^− 1^. The seedlings were thawed at 4 °C for 12 h and then recovered for 3 d at 30 °C. Seedlings, which were not subjected to cold and freezing treatments, served as the controls. There were 16 seedlings for each treatment.

### The chlorophyll fluorescence parameters

Four seedlings were selected from each treatment, and one leaf was sampled from each seedling. Fluorescence parameters were tested at indoor temperature using a *chlorophyll* fluorometer (PAM-2500, Walz, Germany). Seedlings were dark-adapted for 30 min before measurements were conducted. The maximum quantum yield of photosystem II (PSII) (Fv/Fm), effective quantum yield of PS II (Y(II)), photochemical quenching coefficient (qP), non-photochemical quenching coefficient (NPQ), non-regulated (Y(NO)) and regulated (Y(NPQ)) non-photochemical energy loss in PS II as well as relative electron transport rate (rETR) were measured.

### Sample preparation and lipid extraction

Lipids were extracted according to MTBE method. Briefly, samples were first spiked with appropriate amount of internal lipid standards and then homogenized with 200 μL water and 240 μL methanol. After that, 800 μL of MTBE was added and the mixture was ultrasound 20 min at 4 °C followed by sitting still for 30 min at room temperature. The solution was centrifuged at 14000 g for 15 min at 10 °C and the upper organic solvent layer was obtained and dried under nitrogen. Five seedlings were selected from each treatment, and one leaf was sampled from each seedling for lipid extraction.

### LC-MS/MS method for lipid analysis

Reverse phase chromatography was selected for LC separation using CSH C18 column (1.7 μm, 2.1 mm × 100 mm, Waters). The lipid extracts were re-dissolved in 200 μL 90% isopropanol/ acetonitrile, centrifuged at 14000 g for 15 min, finally 3 μL of sample was injected. Solvent A was acetonitrile–water (6:4, v/v) with 0.1% formic acid and 0.1 Mm ammonium formate and solvent B was acetonitrile–isopropanol (1:9, v/v) with 0.1% formic acid and 0.1 Mm ammonium formate. The initial mobile phase was 30% solvent B at a flow rate of 300 μL/min. It was held for 2 min, and then linearly increased to 100% solvent B in 23 min, followed by equilibrating at 5% solvent B for 10 min.

Mass spectra was acquired by Q-Exactive Plus in positive and negative mode, respectively. ESI parameters were optimized and preset for all measurements as follows: Source temperature, 300 °C; Capillary Temp, 350 °C, the ion spray voltage was set at 3000 V, S-Lens RF Level was set at 50% and the scan range of the instruments was set at m/z 200–1800.

### Identification by lipid search

“Lipid Search” is a search engine for the identification of lipid species based on MS/MS math. Lipid Search contains more than 30 lipid classes and more than 1,500,000 fragment ions in the database. Mass tolerance for precursor and fragment were both set to 5 ppm.

### Calculation of lipid double bond index (DBI) and acyl chain length (ACL)

ACL = (∑[n × mol % lipid])/100, where n was the number of acyl carbons in each lipid molecule; DBI = (∑[N × mol % lipid])/100, where N was the number of double bonds in each lipid molecule [15].

### Statistical analysis

The data were subjected to one-way analysis of variance (ANOVA) with SPSS 15.0. Statistical significance was tested by Fisher’s least significant difference (LSD) method. Comparisons between two species were evaluated by T-test.

## Supplementary Information

**Additional file 1: Table S1.** All the identified lipid species in leaves of *C. bifida* and *C. panzhihuaensis*. **Table S2.** The saccharolipids-glycerophospholipids ratio of *C. bifida* and *C. panzhihuaensis* treated at low temperature. **Table S3.** The contents of phosphatidylinositol (PIP) and cardiolipin (CL) in leaves of *C. bifida* and *C. panzhihuaensis* treated at low temperatures. **Table S4.** The double bond index (DBI)-acyl chain length (ACL) ratio of total glycerophospholipids and total main membrane lipids in *C. bifida* and *C. panzhihuaensis* treated at low temperatures. **Table S5.** The diacylglycerol (DAG)-triacylglycerol (TAG) ratio in leaves of *C. bifida* and *C. panzhihuaensis* treated at low temperatures.

## Data Availability

The datasets used and/or analyzed during the current study are available from the corresponding author on reasonable request.

## Abbreviations

ACL: Acyl chain length
CL: Cardiolipin
DAG: Diacylglycerol
DBI: Double bond index
DGDG: Digalactosyldiacylglycerol
Fv/Fm: The maximum quantum yield of photosystem II (PSII)
MGDG: Monogalactosyldiacylglycerol
MGMG: monogalactosylmonoacylglycerol
NPQ: Non-photochemical quenching coefficient
PA: Phosphatidic acid
PC: Phosphatidylcholine
PE: Phosphatidylethanolamine
PG: Phosphatidylglycerol
PI: Phosphatidylinositol
PIP: Phosphatidylinositol phosphate
PS: Phosphatidylserine
qP: Photochemical quenching coefficient
rETR: Relative electron transport rate
SQDG: Sulphoquinovosyldiacylglycerol
TAG: Triacylglycerol
Y(II): Effective quantum yield of PS II
Y(NO): Non-regulated non-photochemical energy loss in PS II
Y(NPQ): Regulated non-photochemical energy loss in PS II
